# Social and structural barriers for adherence to methadone maintenance treatment among Vietnamese opioid dependence patients

**DOI:** 10.1371/journal.pone.0190941

**Published:** 2018-01-18

**Authors:** Bach Xuan Tran, Long Hoang Nguyen, Tung Thanh Tran, Carl A. Latkin

**Affiliations:** 1 Institute for Preventive Medicine and Public Health, Hanoi Medical University, Hanoi, Vietnam; 2 Johns Hopkins Bloomberg School of Public Health, Baltimore, Maryland, United States of America; 3 School of Medicine and Pharmacy, Vietnam National University, Hanoi, Vietnam; 4 Institute for Global Health Innovations, Duy Tan University, Da Nang, Vietnam; Thai Red Cross AIDS Research Centre, THAILAND

## Abstract

**Introduction:**

Methadone maintenance treatment (MMT) services may reduce the risk of HIV transmission if patients completely adhere to the treatment. Identifying adherence patterns and potential related factors is vital for the sustainability of MMT program in Vietnam. This study examined social and structural factors associated with adherence to MMT among patients in different service delivery models.

**Materials and methods:**

A total of 510 patients at three MMT clinics in Hanoi were interviewed. Measures of self-reported adherence included the number of missed doses in the past 7 days and the level of adherence in the past 30 days using a visual analog scale (VAS) scoring from 0 (non-adherence) to 100 (perfect adherence). Multivariate regressions were employed to identify factors associated with non-adherence to MMT.

**Results:**

A total of 17.7% of participants reported incomplete MMT adherence in the last 30 days and 8.3% reported missing a dose in the last seven days, respectively. Living with HIV/AIDS, poor self-care and usual activities, and disclosure of health issues to spouses or intimate partners were associated with non-adherence. Those patients with pain or depression were more likely to report better adherence. Disclosing health status to spouse/partner increased the risk of incomplete adherence, while disclosing to friends reduced the number of missed dose in the last seven days. Patients attending clinics with comprehensive services had a lower VAS score of adherence compared to those enrolling in clinics with only MMT and general health care.

**Conclusions:**

Sustaining the compliance of patients to MMT is principal in the rapid expansion of this service in Vietnam. It is necessary to address the complexity of health care demands of drug users, their difficulties to be rehabilitated into workforce and society, and the stigmatization to maximize the outcomes of MMT program.

## Introduction

Globally, methadone maintenance treatment (MMT) is recognized as an essential and cost-effective substitution therapy for opioid dependent individuals [1–3]. MMT is efficacious in reducing drug use-related consequences and improving health and socioeconomic status of drug users [1,2,4–6]. In injection-driven HIV epidemics, the expansion of MMT services not only reduced the risk of HIV transmission, but also supported patients’ access, utilization and outcomes of other HIV-related services [7–9]. Literature has shown that MMT clinics could be strategic sites in providing directly administered antiretroviral therapy models for HIV-positive drug users [10]. Given its benefits, the expansion of MMT is a vital component of global HIV/AIDS prevention strategies [3,11].

MMT is a long-term and slow-onset substitution therapy, which needs complete medication adherence to achieve optimal outcomes [12,13]. The current practice in Vietnam requires patients to visit clinic daily and take medication under strict supervisions of health staff. This practice is also applied in other South Asia countries such as Bangladesh, India, Nepal and Maldives [14], or in some European countries and Canada [15,16]. In China, in addition to the traditional delivery model, the Government implements a mobile service model to provide methadone for patients in rural and remote areas [17].

Ensuring patients’ adherence has increasingly challenged HIV programs in many settings. In some countries such as China, Vietnam and Malaysia, where concurrent epidemics of HIV and substance abuse exists, it has been well documented that patients’ adherence to MMT was not optimal and the retention rate was just 40% over 3 years [18–20]. In developed settings, a growing body of evidence has shown the poor adherence among patients receiving MMT. In the United States, studies have found 17% of patients did not adhere the therapy, and in Canada this number has been reported to be 16% [21,22]. Other observations in the United Kingdom, France, and Australia indicated high rates of non-adherence at 42.0%; 65.2%, and 33%, respectively [19,23–25].

Several studies have documented individual and biological factors which were associated with non-adherence to MMT services. They included lower socioeconomic status, lack of social support, discrimination and insufficient methadone doses [26–30]. Meanwhile, some structural barriers were also recognized to predict non-adherence to MMT including patients’ satisfaction with healthcare services, poverty, jobs and housing, in both developed and developing countries [19,23,24,28,31–33]. However, these factors varied across study settings, and very few ones have focused on the provision of MMT in relation to other HIV services.

Vietnam is one among those countries with a strong political will to implement MMT program and halt the spread of HIV epidemics in drug-using populations. Since its first introduction in 2008, 251 MMT clinics have been established and providing treatment for over 46,000 patients [34]. The Vietnam’s MMT program has been proved to effectively contribute to the control and prevention of HIV/AIDS [35–37]. However, poor medication adherence poses a great challenge to health managers in the rapid expansion of MMT services in Vietnam. A longitudinal study in Hai Phong and Ho Chi Minh cities in Vietnam showed that after 24 months of treatment, 41.4% patients reported missing dose for 1–2 days [18]. Another study conducted in a mountainous area indicated that 65.6% patients had sub-optimal adherence to MMT [38].

Hanoi is a capital of Vietnam, where is an HIV/AIDS epicenter with more than 18,000 people living with HIV [39] and about 5,000 patients participating in the MMT program [34]. Evidence about the adherence pattern among MMT patients in this city would contribute substantially to the development of strategies to maximize the outcomes of MMT program in Vietnam. Therefore, this study assessed patients’ adherence to MMT services and examined its social and structural determinants.

## Material and methods

### Study design and setting

We conducted a facility-based cross-sectional survey in Hanoi from April to August 2013. We selected clinics based on following criteria: 1) Currently providing the MMT service, and 2) Having at least 100 patients taking MMT during data collection. Among six eligible facilities, we randomly chose three clinics including: Tu Liem district health center (DHC), Long Bien DHC and Ha Dong polyclinic. The features of these clinics are described in **Table 1**.

**Table 1:** Study settings and sample size.

Level	Settings	Site Name	Type of services	Sample size
District (urban)	Tu Liem District	District Health Centre	MMT+ HCT + ART + GH*	201
District (urban)	Long Bien District	District Health Centre	MMT+ HCT + ART + GH*	99
District (urban)	Ha Dong District	Regional Polyclinic	MMT+ GH*	210

* MMT: Methadone maintenance treatment; HCT: HIV counseling and testing; ART: antiretroviral therapy; GH: General health care

### Sample size and sampling method

We used a convenience sampling technique to recruit patients. All patients at these selected clinics were offered invitations to participate in the study if they were receiving MMT at the selected clinics, 18 years old or above, and attended clinics during the study period. Finally, data from 510 patients were used for analysis (response rate 80–90% across clinics).

### Measures and instruments

Data were obtained via face-to-face interviews using a structured questionnaire. A private room was employed for the interviews to assure the confidentiality of patients. Interviewers were students in Master of Public Health program at Hanoi Medical University, under the supervision of experts in the field of substance abuse. Each interview was performed in 15–20 minutes.

The main outcome of this study was the self-reported medication adherence. Patients were asked to report their adherence in the last seven days by using the question: “How many days did you miss dose in the last seven days?”. Optimal adherence was detected when patients did not miss any dose. Moreover, a 100-point visual analog scale (VAS) was also employed to detect patients’ adherence in the last 30 days, with a score range from 0 “complete non-adherence” to 100 “perfect adherence”. These approaches had been successfully applied in a previous study [38]. While the number of missed doses in the past 7 days is an indicator to describe recent adherence, the VAS score is a general measure that reflects the overall adherence over a period of treatment. These two indicators were supplementary to each other. We also asked patients to report their reasons if they did not adhere the treatment.

In this study, based on previous literatures, we measured social and structural factors as below:

#### Socioeconomic status

We collected socio-demographic status of patients, including: age, gender, education, marital status and employment status.

#### Social factors

**Health status.** We applied a well-validated tool namely EuroQol—five dimensions–five levels (EQ-5D-5L), which evaluated five components: Mobility, Self-care, Usual activities, Pain/Discomfort and Anxiety/Depression. Each domain had five response levels from no problem to extreme problems. This instrument was used previously in the context of Vietnam [5,38,40–49].

**Stigmatization.** We investigated the drug use-related stigma among patients by using the instrument that was used and validated elsewhere [50,51]. We evaluated four aspects of stigma including: (1) Blame/Judgement, (2) Shame, (3) Discrimination in several settings (work place, health care services, family, and community), (4) Disclosure of addiction or health status. The short description of this instrument can be found in our previous publications [50,51]. In short, patients were asked to report whether they suffered any of these kinds of stigma in the last 30 days.

#### Structural factors

**Satisfaction.** We also employed an instrument entitled the Satisfaction with HIV/AIDS Treatment Interview Scale (SATIS), which was used and validated previously in Vietnam to measure the satisfaction of patients to the HIV-related services such as ART and MMT [52,53]. Overall, the SATIS has ten items with a score range from 0 “complete dissatisfaction” to 10 “complete satisfaction”. There are three sub-domains namely: “Services quality and convenience”, “Capacity health workers & responsiveness” and “Inter-professional care”. The score of each sub-domain was calculated by averaging the score of all items in the sub-domains [52,53].

### Statistical analysis

STATA software version 12.0 (Stata Corp. LP, College Station, United States of America) was used to analyze data. A *p*-value <0.05 was used to detect statistical significance. T-test, Mann-Whitney test, Chi-square test and Fisher’s exact test were used to assess the differences of variables between adherence and non-adherence groups. We utilized multivariate Logistic, Tobit and Zero-inflated Poisson regressions to identify factors associated with MMT adherence among patients. A step-wise forward selection strategy was used with a *p*-value of <0.2 as a threshold of the log-likelihood ratio test to select variables.

### Ethical consideration

All study materials were approved by the IRB of the Vietnam Authority of HIV/AIDS Control. Data collection procedures were also approved by the directors of each of the three MMT clinics included in the study. Written informed consent was obtained from all participants.

## Results

Among 510 patients, the mean age was 36.6 (SD = 7.7) years old. Most of them were male (98.4%), attaining less than high school education (53.7%) and living with their spouse/partners (70.0%). The majority of respondents were self-employed (52.7%) or unemployed (26.6%). Compared with the incomplete adherence group, more respondents in the complete adherence group had under high school education (56.2% vs 41.3%) (p = 0.03). We did not find any differences in other socioeconomic and health status factors (**Table 2**).

**Table 2:** Medication adherence by socioeconomic characteristics among MMT patients.

Characteristics	Complete adherence		Incomplete adherence		Total		OR (95%CI)	p-value
	n	%	n	%	n	%		
**Total**	417	82.2	90	17.8	507	100.0		
**Age (years), mean, SD**	36.8	7.6	35.5	7.8	36.6	7.7	0.98 (0.95–1.01)	0.05
**Gender**								
Male	411	98.3	91	98.9	502	98.4	1.00 (ref)	0.56
Female	7	1.7	1	1.1	8	1.6	0.65 (0.08–5.31)	
**Education**								
< High school	235	56.2	38	41.3	273	53.5	1.00 (ref)	0.03
High school	155	37.1	45	48.9	200	39.2	1.80 (1.11–2.89)	
> High school	28	6.7	9	9.8	37	7.3	1.99 (0.87–4.54)	
**Marital status**								
Single	83	19.9	23	25.0	106	20.8	1.00 (ref)	0.17
Live with spouse/partner	294	70.3	65	70.7	359	70.4	0.80 (0.47–1.36)	
Divorced/Separate/Widow	41	9.8	4	4.3	45	8.8	0.35 (0.11–1.09)	
**Employment**								
Unemployed	112	26.8	23	25.0	135	26.5	1.00 (ref)	0.35
Freelancer	222	53.0	47	51.1	269	52.8	1.03 (0.60–1.78)	
Stable jobs	42	10.1	7	7.6	49	9.6	0.81 (0.32–2.03)	
Other jobs	42	10.1	15	16.3	57	11.2	1.74 (0.83–3.65)	

OR: Odds ratio; ref: reference

**Table 3** presents the adherence patterns among respondents. About 91.7% patients did not miss dose in the last seven days and 82.3% reported complete adherence in the last 30 days. Having busy work was the primary reason for non-adherence with 57.1%, following by not remembering to take doses (11.9%).

**Table 3:** Medication adherence among MMT patients.

	n	%
**Missed dose in 1 week**		
Not missed	465	91.7
Missed	42	8.3
Complete adherence (VAS*) in the last 30 days		
Complete Adherence	417	82.2
Incomplete Adherence	90	17.8
**Reason of non-adherence (n = 42)**		
Long distance from home	3	7.1
Forget	5	11.9
Busy work	24	57.1
Having illness	3	7.1
Unknow	3	7.1
Others	3	7.1
	**Mean**	**SD**
VAS* score for adherence	92.5	24.2

* VAS: Visual analogue scale.

**Table 4** indicates that in comparison with the incomplete adherence group, a lower proportion of patients had usual activity problems in the complete adherence group (p = 0.04). Moreover, a larger proportion of incomplete adherers disclosed health status to their spouse/partner compared to that of complete adherers (p = 0.04). Meanwhile, all other factors were not found to be significantly different between the complete and incomplete adherence groups.

**Table 4:** Differences of social factors between adherence and non-adherence among MMT patients.

Characteristics	Complete adherence		Incomplete adherence		Total		OR ^a^ (95%CI)	p-value
	n	%	n	%	N	%		
**HIV positive**	32	7.7	13	14.1	45	8.8	1.98 (1.00–3.95)	0.13
**Health status**								
Having problems in mobility	22	5.3	8	8.7	30	5.9	1.71 (0.74–3.98)	0.21
Having problems in self-care	14	3.4	5	5.4	19	3.7	1.66 (0.58–4.73)	0.34
Having problems in usual activities	13	3.1	7	7.6	20	3.9	2.57 (0.99–6.62)	0.04
Pain or Discomfort	69	16.5	10	10.9	79	15.5	0.62 (0.30–1.25)	0.18
Anxiety or Depression	73	17.5	11	12.0	84	16.5	0.64 (0.33–1.26)	0.20
**Feeling blame/judge**	54	13.0	13	14.3	67	13.2	1.12 (0.58–2.15)	0.74
**Feeling shame**	48	11.6	12	13.3	60	11.9	1.17 (0.60–2.31)	0.64
**Feeling discrimination in**								
Workplace	3	0.7	1	1.1	4	0.8	1.52 (0.16–14.78)	0.72
Healthcare service	2	0.5	1	1.1	3	0.6	2.29 (0.21–25.48)	0.49
Family	4	1.0	2	2.2	6	1.2	2.30 (0.41–12.75)	0.33
Community	30	7.2	8	8.7	38	7.5	1.23 (0.55–2.78)	0.62
**Disclose health status to**								
Spouse/Partner	251	60.1	66	71.7	317	62.2	1.69 (1.03–2.77)	0.04
Parents	205	49.0	53	57.6	258	50.6	1.41 (0.90–2.23)	0.14
Other relatives	130	31.1	26	28.3	156	30.6	0.87 (0.53–1.44)	0.59
Friends	115	27.5	28	30.4	143	28.0	1.15 (0.70–1.89)	0.57
Health staffs	171	40.9	44	47.8	215	42.2	1.32 (0.84–2.08)	0.22
Peers	54	12.9	17	18.5	71	13.9	1.53 (0.84–2.78)	0.16
	**Mean**	**SD**	**Mean**	**SD**	**Mean**	**SD**		
Duration of MMT (month)	20.4	11.9	22.2	12.0	20.7	11.8	1.01 (0.99–1.03)	0.14

OR: Odds ratio;

^a^ Reference group: Not have these health states or experience any stigmatizations

**Table 5** indicates that there was not any difference found between the adherence and non-adherence groups regarding structural factors such as MMT delivery models and the satisfaction of patients.

**Table 5:** Differences of structural factors between adherence and non-adherence among MMT patients.

Characteristics	Complete adherence		Incomplete adherence		Total		OR (95%CI)	p-value
	n	%	n	%	n	%		
**MMT delivery models**								
MMT+ HCT + ART + GH*	236	56.6	61	67.8	297	58.6	1.00 (ref)	0.051
MMT+ GH*	181	43.4	29	32.2	210	41.4	1.61 (0.99–2.61)	
	**Mean**	**SD**	**Mean**	**SD**	**Mean**	**SD**		
**Satisfaction with services**								
Services quality and convenience	9.2	1.1	9.3	0.99	9.25	1.07	1.09 (0.87–1.38)	0.24
Capacity health workers & responsiveness	9.2	1.3	9.4	1.03	9.24	1.26	1.10 (0.89–1.35)	0.24
Inter-professional care	9.3	1.1	9.4	0.98	9.29	1.10	1.09 (0.87–1.38)	0.28
Overall SATIS Score	9.3	1.1	9.35	0.97	9.26	1.07	1.11 (0.87–1.41)	0.30

* MMT: Methadone maintenance treatment; HCT: HIV counseling and testing; ART: antiretroviral therapy; GH: General health care; OR: Odds ratio; ref: reference

**Table 6** shows the results of reduced multivariate regressions. Higher age, having high school degree, HIV positive and disclosing health status to spouse/partner were more likely to increase the number of missed doses. Meanwhile, people having anxiety/depression and disclosing health status to friends had a lower number of missed doses than others. In addition, higher age (OR = 0.94; 95%CI = 0.90–0.99) were associated with lower likelihood of missing dose, while having problems in self-care was a significant risk factor for missing dose (OR = 4.47; 95%CI = 1.31–15.23).

**Table 6:** Multivariate regressions to identify factors associated with non-adherence among MMT patients.

	No. of missed dose in the last 7 days		Ever missed dose in the last 7 days		VAS Score of adherence		Incomplete adherence (VAS)	
	Coef.	95%CI	OR	95% CI	Coef.	95%CI	OR	95% CI
**Age** (years)	0.07***	0.03; 0.10	0.94**	0.89; 0.99			0.97*	0.94; 1.01
**Education** (vs < High school)								
High school	0.77**	0.12; 1.41			-27.39*	-55.13; 0.36	1.78**	1.08; 2.95
> High school	0.21	-0.77; 1.19			-48.82*	-100.03; 2.38	1.86	0.76; 4.54
**Marital status** (vs Single)								
Divorce/Separate/Widow	-0.25	-1.14; 0.64	0.23	0.03; 1.76	60.96*	-3.35; 125.27	0.40	0.12; 1.39
**Occupation** (vs Unemployed)								
Stable jobs	-0.48	-1.10; 0.13			42.26	-9.40; 93.93		
**HIV status** (vs Negative)								
Positive	2.47***	1.33; 3.61			-42.05*	-85.52; 1.42	1.89	0.86; 4.17
Unknown	1.01*	-0.17; 2.18						
**Having problems in self-care** (Yes vs No)			4.94***	1.54; 15.80				
**Having problems in usual activities** (Yes vs No)					-69.46**	-135.10; -3.81	3.74**	1.18; 11.81
**Pain/Discomfort** (Yes vs No)	0.82*	-0.01; 1.64			60.08**	13.67; 106.49	0.43**	0.18; 1.00
**Anxiety/Depression** (Yes vs No)	-2.22***	-3.44; -1.00						
**Feeling discrimination in workplace** (Yes vs No)			5.48	0.48; 62.85				
**Feeling discrimination in health service** (Yes vs No)			13.31*	0.81; 219.13				
**Feeling discrimination in community** (Yes vs No)	-0.45	-1.92; 1.01						
**Disclose health status to Spouse/Partner** (Yes vs No)	1.79***	0.85; 2.74			-26.11*	-57.15; 4.94	1.90**	1.07; 3.37
**Disclose health status to Other relatives** (Yes vs No)							0.67	0.37; 1.23
**Disclose health status to Friends** (Yes vs No)	-0.90**	-1.62; -0.18						
**Disclose health status to Health staffs** (Yes vs No)					-19.31	-47.29; 8.68		
**Disclose health status to Peers** (Yes vs No)	-1.21	-3.07; 0.65	0.34	0.08; 1.49			1.70	0.82; 3.52
**MMT delivery models** (MMT+HTC+ART+GH vs MMT+GH)	0.76*	-0.14; 1.65	1.86*	0.89; 3.88	-29.03**	-56.81; -1.24	1.55*	0.93; 2.60
**Services quality and convenience**	-0.57*	-1.23; 0.10	0.61	0.32; 1.15				
**Capacity health workers & responsiveness**	0.80*	-0.13; 1.74	1.73*	0.92; 3.25	-11.89*	-24.23; 0.46		
**Inter-professional care**	0.28	-0.37; 0.93						

*** p<0.01.

** p<0.05.

* p<0.1.

Regarding the VAS score of adherence, patients having pain/discomfort had higher score of adherence than those not suffering. Having problems in usual activities and attending clinics with full services (MMT+ART+HTC+GH) were negatively associated with the VAS score of adherence. People having high school education, having problems in usual activities and disclosing health status to spouse/partner were more likely to incompletely adhere the medication, while respondents having pain/discomfort were less likely to be incomplete adherers.

## Discussion

Although complete adherence to MMT is principal in ensuring drug abstinence, we have found that over one sixth of the study sample reported incomplete adherence. This was driven by the complexity of health care demands and the difficulties constraining drug users in the reintegration into workforce and society.

The proportion of MMT non-adherence in this study (17.8%) was lower than observations in other studies across diverse settings, such as in France (42,0%) [24]; the United Kingdom (65.2%) [23]; Australia (33.0%) [25] and China (36.3% to 88.2%) [19,31]. We would acknowledge the potential reason for this difference that we measured adherence using missed doses in the past 7 days and overall VAS scores for the past 30 days; meanwhile, other researchers assessed adherence over 1, 3 and 12 months. In addition, some patients may over-report the level of adherence using VAS while ignoring doses they have missed if it was too few.

Comparing to the previous study in Vietnam, the proportion of 8.3% patients missed doses in the past seven days in our observation is lower than that in a mountainous setting in Vietnam (10%) [38]. Urban patients probably access more easily to MMT clinics than those with geolocation barriers. Noticeably, in mountainous settings, the 3-month non-adherence rate was 65.6% and increased over the course of MMT [38]. Therefore, the adherence monitoring and the provision of support and counseling should be maintained to reassure the MMT retention and complete adherence of the patients [18,38].

In this study, higher age was found to be associated with better treatment adherence, while high education was related to the incomplete adherence. These results are similar to other previous findings [19,54,55]. Besides, patients having problems in self-care had a higher chance to be non-adherers. Noteworthy is that poor self-care might restrict the functional capacity of patients and reduce the effort to visit the clinic for taking medicine [56]. Otherwise, patients having pain/discomfort were more likely to adhere the medication, which was contrary to the previous findings that having physical and psychological problems could decrease adherence [24,38]. Notably, the main reason for not adherence was having busy work (57.1%). Since most of the respondents were self-employed or worked for hire with daily wages, they have to manage their time and commitment to their work as well as to go to the clinic for MMT [19,57,58]. Poverty and job commitment have been described as major structural barriers for adherence to healthcare services in many settings. In this study, we enrich the literature by describing the concurrent impacts of job commitment and stigma to drug users. Having a job is very necessary for the patients by improving their social and economical status, but their physical healthcare was demanding the methadone medications daily. This is also an explanation that those with better health status will go for work and poorly adhered to MMT, while those with severer pain or depression issues were better adhering to the treatment.

Interestingly, we found that people who ever disclosed to their spouse/partner were more likely to report incomplete adherence, while patients disclosing their status to their friends were more likely to adhere the medication. Literatures emphasized the benefits of health status disclosure to medication adherence in various patient groups such as having more social support, less psychological distress and receiving timely coping strategies for their problems [30,59,60]. However, these advantages rely on how the patients interact with their societies [61,62]. If they had negative relationships, disclosure could lead to serious stigmatization and rejection, and eventually, negatively influence medication adherence [30]. Besides, only a few patients reported feeling stigma in their workplaces, families, health services or communities. Perhaps, patients who disclosed their health status to the spouse/partner could release the pressure on themselves regarding treatment, and tend to allow them skipping several doses without any concerns about clinical consequences [30,63]. It could also be the case that their spouse/ partner was also a drug abuser that make it more difficult for the patient to completely adhere to MMT.

When we investigated structural barriers such as MMT delivery models and the satisfaction of patients to the adherence, we only found that patients attending clinics delivering comprehensive services (MMT+ART+HCT+GH) had a lower VAS score of adherence compared to those enrolling in clinics with only MMT and GH. However, rather than the provision of services, this could be explained by potential interactions of ART and methadone, which was found to be related with lower methadone serum concentrations and adverse effects such as depression, insomnia and myalgias, facilitating the non-adherence among HIV(+) patients (as shown in multivariate regression) [64–66].

The findings of this study suggest some implications. First, the participation of patients’ spouse or partners has a central role in reminding patients to adhere the medication. Second, MMT clinics should be integrated with the general health care service in order to understand the needs of health care among patients and resolve timely during their treatment. Moreover, the provision of MMT program should be connected among clinics, which can help patients to access MMT in the nearest places where they feel convenient to take medication. Finally, developing early warning system involving both clinical and self-reported data to inform the adherence of patients might be helpful to improve the treatment outcome of MMT program.

There are several limitations in this study that should be recognized. First, using the cross-sectional design did not permit us to understand the causal associations between adherence and its determinants. Second, we only collected self-reported information, which might lead to recall bias [67]. Finally, our small sample size recruited by the convenience sampling method might limit the generalizability of this study to other MMT populations. Moreover, medication adherence is complicated to measure by self-reported data alone; therefore, a larger survey with more clinical, biomedical and behavioral information should be warranted in the future to provide the comprehensive view of this issue.

## Conclusion

In conclusion, our study showed a low rate of non-adherence among MMT patients in a Vietnam metropolitan compared to other settings. Having stable jobs, improve the ability to engage in self-care, promote the role of spouse/partner and friends of patients, integrating MMT clinics with general health care and connecting clinics in the provision of MMT program could be potential solutions to enhance the adherence of MMT patients.
